# Supplementary material for: Intercellular network structure and regulatory motifs in the human hematopoietic system
Source: Mol Syst Biol. 2014 Jul 15;10(7):741. doi: 10.15252/msb.20145141 (PMC4299490; doi:10.15252/msb.20145141)
Supplement: Supplementary file 1 — Supplementary Figure S1 [file msb0010-0741-sd1.pdf]

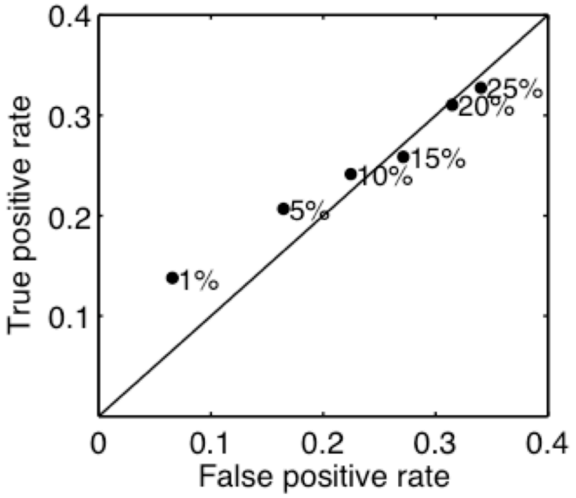

Figure S1. Receiver operating characteristic curve for the identified differentially over-expressed receptor genes at varying false discovery rates (1%, 5%, 10%, 15%, 20% and 25%) for definition of differential over-expression. See "identification of differentially over-expressed genes" in the Materials and Methods for the list of cell type-specific receptors that were used as true positives in this analysis. Related to Figure 2.
