# Supplementary material for: Intercellular network structure and regulatory motifs in the human hematopoietic system
Source: Mol Syst Biol. 2014 Jul 15;10(7):741. doi: 10.15252/msb.20145141 (PMC4299490; doi:10.15252/msb.20145141)
Supplement: Supplementary file 2 — Supplementary Figure S2 [file msb0010-0741-sd2.pdf]

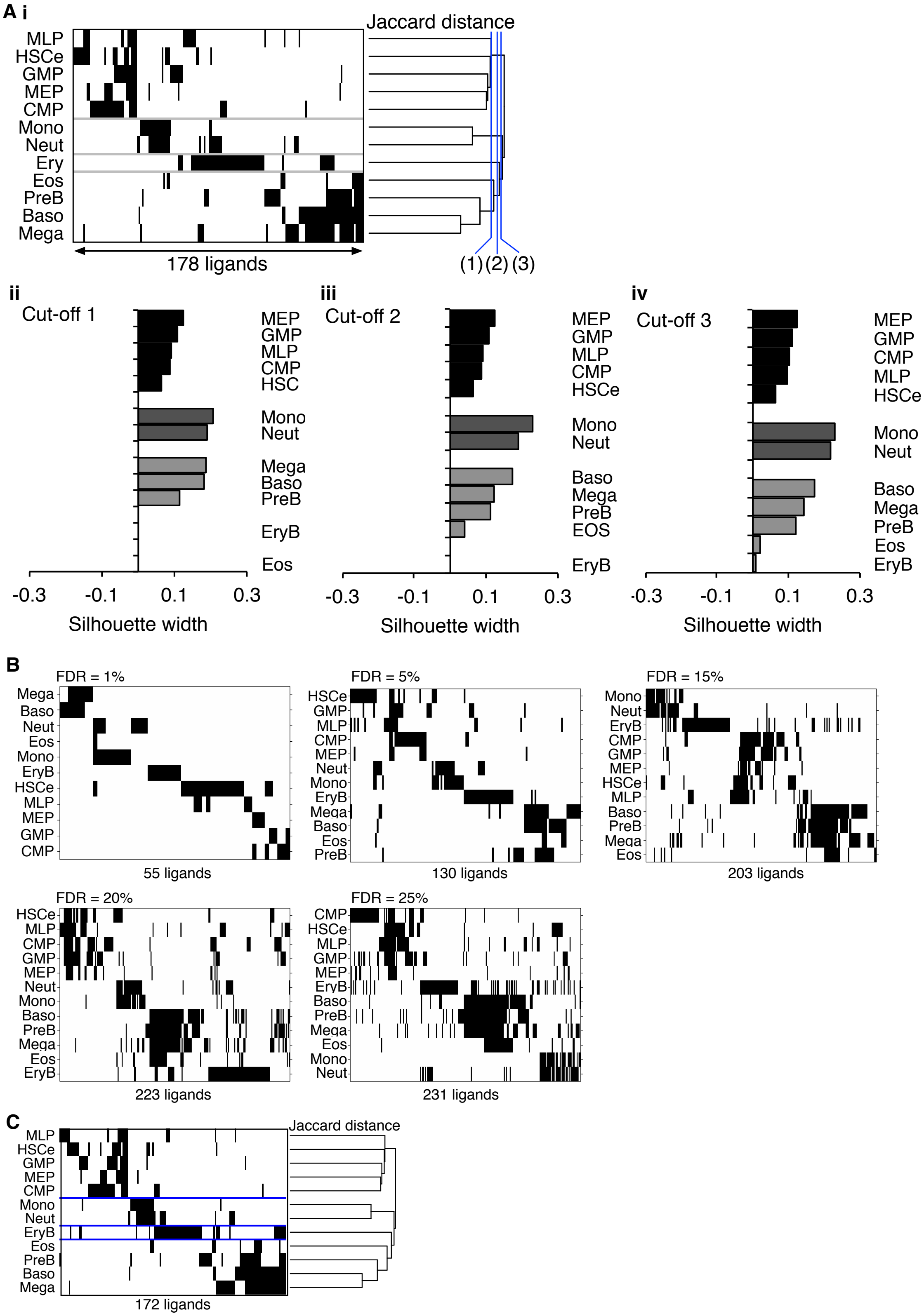

Figure S2. Modular structure in the ligand production network.

A Cell modules in terms of ligand production. (i) Jaccard distance-based average hierarchical clusters for the cell-to-ligand interaction in the ligand production network constructed at false discovery rate (FDR) of 10%. The cell types in the module of primitive cells (HSCe + MLP + GMP + MEP + CMP) were not separable because the heights of the linkages were similar. Using the height of the primitive cell module as a reference, modules of (Mono + Neut) and (PreB + Baso + Mega) were obvious. To classify EryB and Eos, modules obtained at three Jaccard distance cutoffs were quantified. (ii) Silhouette widths for the five modules defined at cut-off 1. (iii) Silhouette widths for the four modules defined at cut-off 2. This result is further discussed in Figure 3 because EryB expressed a group of unique ligands comparing to the other cell types. (iv) Silhouette width for the 3 modules defined at cut-off 3. Regardless the cut-off values, cell types within each module exhibited non-negative Silhouette width.

B Jaccard distance-based average hierarchical clustering for the cell-to-ligand interaction in the ligand production network. The analysis was performed for the networks constructed at FDRs of 1%, 5%, 15%, 20% and 25%. This result suggests that modularity in the ligand production network is robust to network size. Related to Figure 3.

C Jaccard distance-based average hierarchical clusters for the cell-to-ligand interaction in the ligand production network (FDR = 10%) after incorporation of hetero-multimeric receptors into network construction. For class-1 cytokine ligands, the interaction between a ligand and a cell establishes only if both the ligand binding arm(s) and the signaling arm of a hetero-multimeric receptor are differentially over-expressed in a cell type. For this reason, six ligands in the network shown in Figure S2A-i, including OSM (produced by GMP and EryB), IL7 (produced by MEP, Mega and Baso), IL15 (produced by Neut and Mono), IL3 (produced by CMP), LIF (produced by Neut and EryB), and CTF1 (produced by EryB) are not in the heteromultimeric receptor-based network.

Related to Figure 3.
