# Supplementary material for: Intercellular network structure and regulatory motifs in the human hematopoietic system
Source: Mol Syst Biol. 2014 Jul 15;10(7):741. doi: 10.15252/msb.20145141 (PMC4299490; doi:10.15252/msb.20145141)
Supplement: Supplementary file 3 — Supplementary Figure S3 [file msb0010-0741-sd3.pdf]

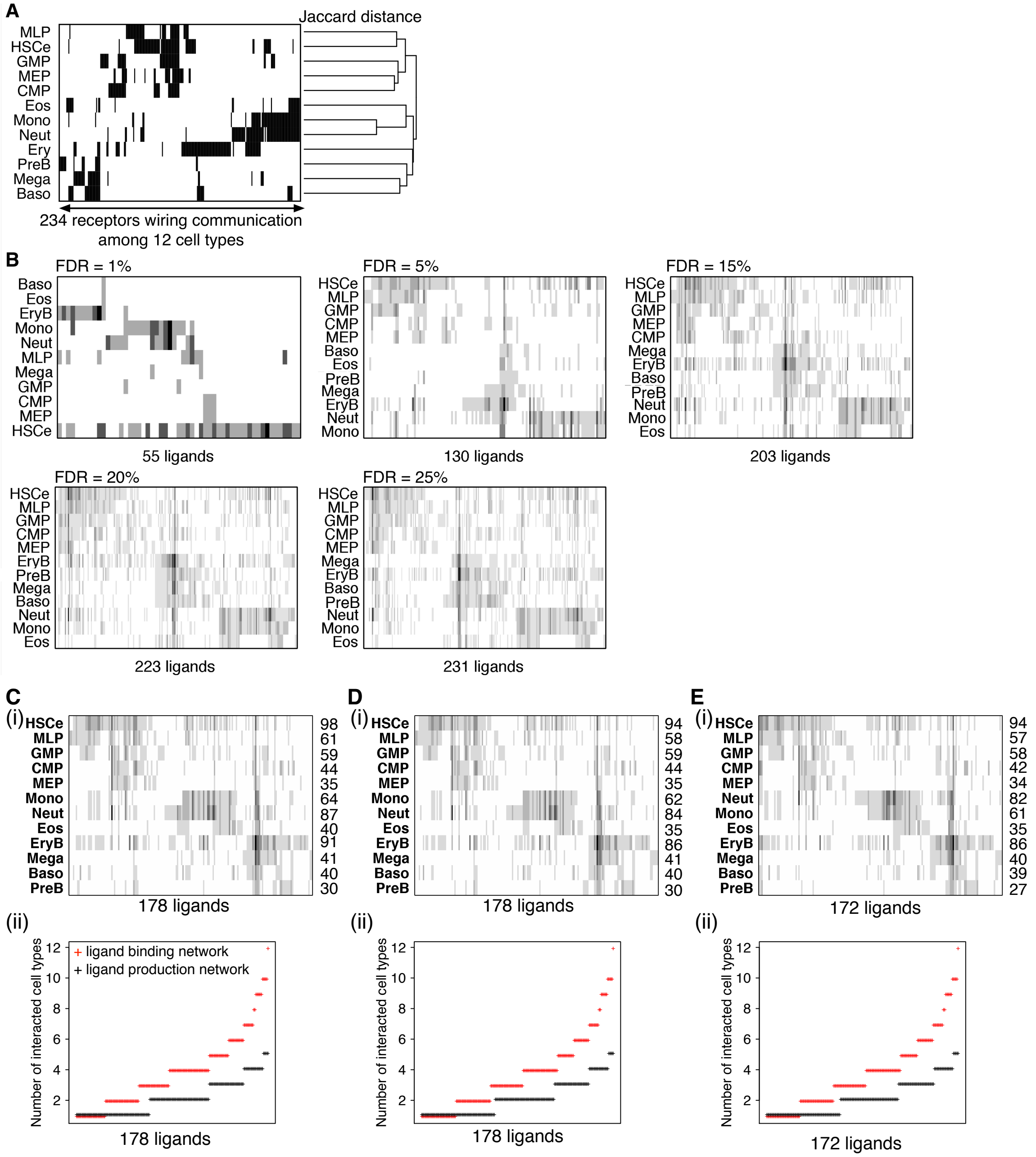

Figure S3. Promiscuous structure in the ligand binding network and its consequent effects on HSCe-targeting signals.

A Receptor expression by the hematopoietic cells of interest shows a modular structure, meaning that promiscuity in the ligand binding network (false discovery rate, FDR = 10%) shown in Figure 4A is the result of non-specific ligand-receptor interaction, i.e., one receptor binds to multiple ligands.

B Spectrum co-clustering of the ligand-to-cell interaction in ligand binding networks constructed at different FDRs, i.e., networks of different sizes. Grey

scales indicate the number of receptors expressed for each ligand. This result suggests that promiscuity in the ligand binding network is robust to network size.

C Network that treats the interactions between a ligand and monomeric receptors independently (FDR = 10%). (i) Spectral co-clustered ligand binding network. The numbers on the right indicate the number of bound ligands of each cell type. (ii) The number of interacted cell types of each ligand in the ligand binding network and in the ligand production network. While ligands in the production networks interacted with at most 5 cell types, whereas certain ligands in the binding networks interacted with up to 12 cell types, implying that the ligand-cell interactions in the binding networks were less modular (i.e., more promiscuous) than the interactions in the production networks.

D Network that counts ligand-to-cell interactions when the ligand binding arm of a heteromultimeric receptor are expressed (FDR = 10%). (i) Spectral co-clustered ligand binding network. (ii) The number of interacted cell types of each ligand in the ligand binding network and in the ligand production network. While ligands in the production networks interacted with at most 5 cell types, whereas certain ligands in the binding networks interacted with up to 12 cell types, implying that the ligand-cell interactions in the binding networks were less modular (i.e., more promiscuous) than the interactions in the production networks.

E Network that count ligand-to-cell interactions when both the binding arm and the signaling arm of a heteromultimeric receptor are expressed (FDR = 10%). (i) Spectral co-clustered ligand binding network. (ii) The number of interacted cell types of each ligand in the ligand binding network and in the ligand production network. While ligands in the production networks interacted with at most 5 cell types, whereas certain ligands in the binding networks interacted with up to 12 cell types, implying that the ligand-cell interactions in the binding networks were less modular (i.e., more promiscuous) than the interactions in the production networks.

Related to Figure 4.
