# Supplementary material for: Intercellular network structure and regulatory motifs in the human hematopoietic system
Source: Mol Syst Biol. 2014 Jul 15;10(7):741. doi: 10.15252/msb.20145141 (PMC4299490; doi:10.15252/msb.20145141)
Supplement: Supplementary file 4 — Supplementary Figure S4 [file msb0010-0741-sd4.pdf]

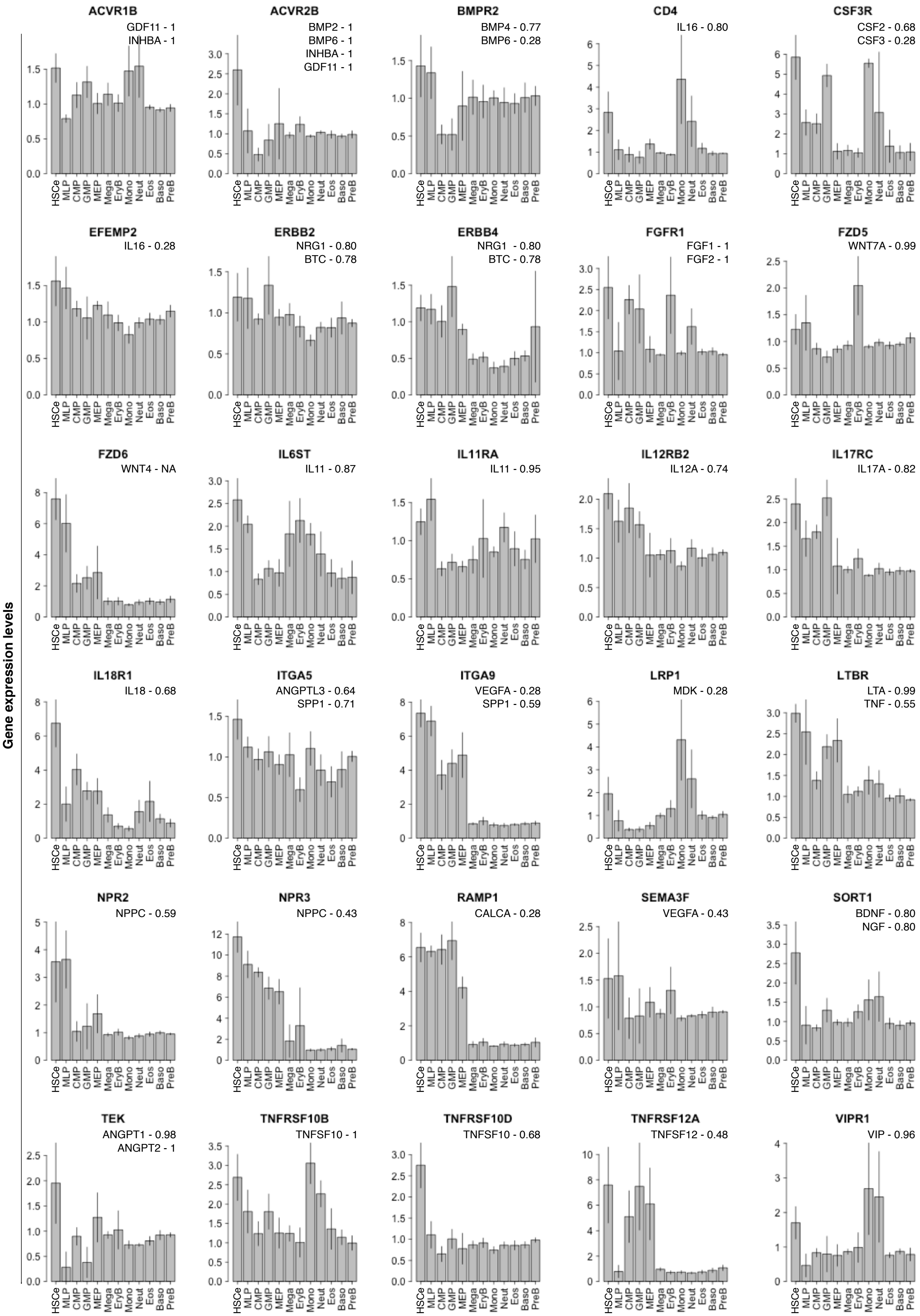
Gene expression levels
ACVR1B
GDF11 - 1
INHBA - 1
ACVR2B
BMP2 - 1
BMP6 - 1
INHBA - 1
GDF11 - 1
BMPR2
BMP4 - 0.77
BMP6 - 0.28
CD4
IL16 - 0.80
CSF3R
CSF2 - 0.68
CSF3 - 0.28
EFEMP2
IL16 - 0.28
ERBB2
NRG1 - 0.80
BTC - 0.78
ERBB4
NRG1 - 0.80
BTC - 0.78
FGFR1
FGF1 - 1
FGF2 - 1
FZD5
WNT7A - 0.99
FZD6
WNT4 - NA
IL6ST
IL11 - 0.87
IL11RA
IL11 - 0.95
IL12RB2
IL12A - 0.74
IL17RC
IL17A - 0.82
IL18R1
IL18 - 0.68
ITGA5
ANGPTL3 - 0.64
SPP1 - 0.71
ITGA9
VEGFA - 0.28
SPP1 - 0.59
LRP1
MDK - 0.28
LTBR
LTA - 0.99
TNF - 0.55
NPR2
NPPC - 0.59
NPR3
NPPC - 0.43
RAMP1
CALCA - 0.28
SEMA3F
VEGFA - 0.43
SORT1
BDNF - 0.80
NGF - 0.80
TEK
ANGPT1 - 0.98
ANGPT2 - 1
TNFRSF10B
TNFSF10 - 1
TNFRSF10D
TNFSF10 - 0.68
TNFRSF12A
TNFSF12 - 0.48
VIPR1
VIP - 0.96
HSCe
MLP
CMP
GMP
MEP
Mega
EryB
Mono
Neut
Eos
Baso
PreB

Figure S4. Receptor gene expression in the blood cell types of interest.

Numerical values in each figure legend are the MINT-inspired scores that measure the confidence (between 0 and 1) in molecular interactions annotated from literature. The data shown are mean ± sd. Related to Figure 5.
