# Supplementary material for: Intercellular network structure and regulatory motifs in the human hematopoietic system
Source: Mol Syst Biol. 2014 Jul 15;10(7):741. doi: 10.15252/msb.20145141 (PMC4299490; doi:10.15252/msb.20145141)
Supplement: Supplementary file 5 — Supplementary Figure S5 [file msb0010-0741-sd5.pdf]

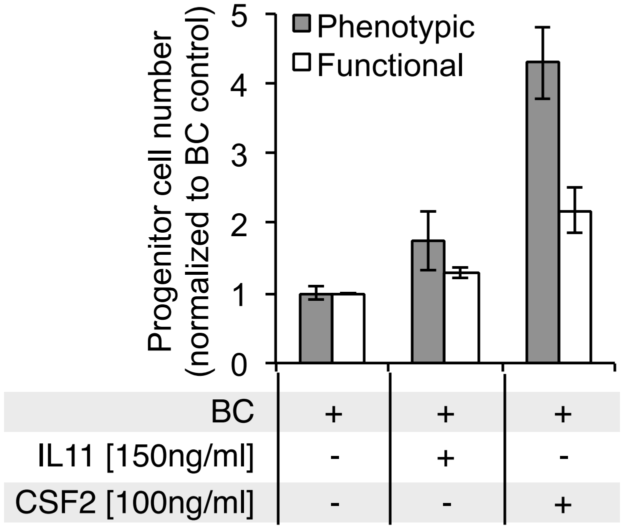

Figure S5. Comparison between progenitor cell counts obtained using a phenotypic assay and the functional colony-forming cell (CFC) assay.

In the *in vitro* study, progenitor cells were defined by the cell surface marker combination ($CD34^{+}133^{-}$ or $CD90^{-}$). Using IL11 and CSF2 as representative ligands, we quantified the outputs of this marker combination by comparing the results to the results of the CFC assay for progenitor cell quantification. The relative progenitor cell counts measured by the surface marker combination correlated with that quantified by the CFC assay. This result provided us confidence of using ($CD34^{+}133^{-}$ or $CD90^{-}$) to quantify progenitor cell outputs of our cell culture. The CFC counts were obtained using the following protocol. Forty HSC-e were cultured in serum free media supplemented with the basal cytokines (BC, 100ng/ml SCF + 100ng/ml FLT3LG + 50ng/ml THPO), BC + 150ng/ml IL11, and BC + 100 ng/ml CSF2. See the "*in vitro* experiments" section in the Materials and Methods for details. On day 7, the culture-derived cells were assayed for CFC frequency by plating into 1.5 ml methylcellulose-based medium (MethoCult H4434, Stem Cell Technologies) in duplicates. After 14-day incubation at 37ºC in humidified atmosphere of 5% CO2 in air, duplicate cultures were visually scored for CFC content. Shown are mean ± std (n = 33 for the phenotypic data of CSF2; n = 4 for the phenotypic data of IL11; n = 2 for the functional data). Related to Figure 5.
