# Supplementary material for: Intercellular network structure and regulatory motifs in the human hematopoietic system
Source: Mol Syst Biol. 2014 Jul 15;10(7):741. doi: 10.15252/msb.20145141 (PMC4299490; doi:10.15252/msb.20145141)
Supplement: Supplementary file 6 — Supplementary Figure S6 [file msb0010-0741-sd6.pdf]

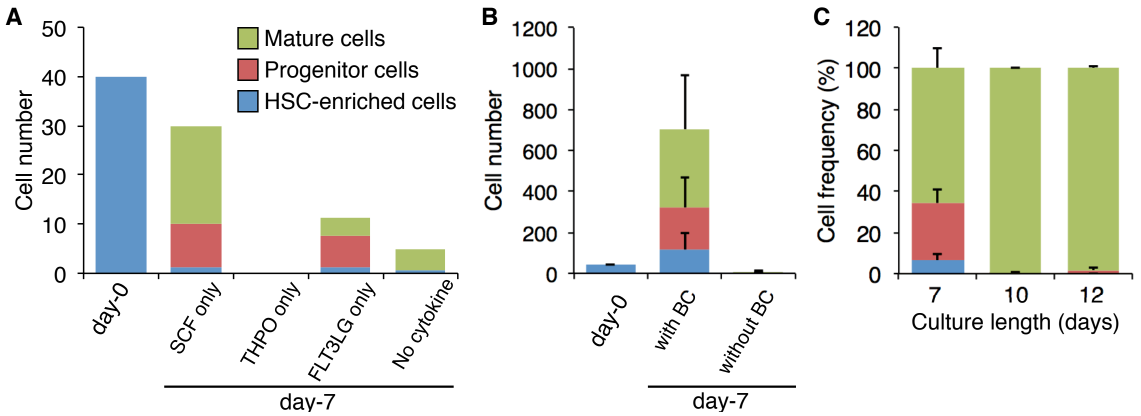

Figure S6. Results of culturing 40 human umbilical cord blood $Lin^{-}Rho^{low}CD34^{+}CD38^{-}CD45RA^{-}CD49f^{+}$ HSC-enriched (HSC-e) cells under different *in vitro* conditions. Due to the low frequency of HSC-e ($Lin^{-}Rho^{low}CD34^{+}CD38^{-}CD45RA^{-}CD49f^{+}$) cells in human umbilical cord blood samples, we decided to seed 40 cells on day 0.

A Results of cell cultures supplemented with different cytokine combinations. day-0: 40 HSC-enriched cells were used to initiate the cell culture; SCF: 100 ng/ml stem cell factor; THPO: 50 ng/ml thrombopoietin; FLT3LG: 100 ng/ml fms-related tyrosine kinase 3 ligand. Green: $CD34^{+}CD133^{+}CD90^{+}$ HSC-enriched cells; red: $CD34^{+}$ progenitor cells that are $CD133^{-}$ or $CD90^{-}$; blue: $CD34^{-}$ mature cells. Data shown are from one experiment.

B Results of cell cultures supplemented with and without basal cytokines (BC = 100 ng/ml SCF + 50 ng/ml THPO + 100 ng/ml FLT3LG). Data shown are mean ± sd (n = 33 biological replicates). Results in A and B demonstrated the need of using the cytokine combination of SCF, THPO and FLT3LG.

C Compositions of BC-supplemented cell cultures on day 7, day 10 and day 12. Data shown are mean ± sd (n = 4 technical replicates). This result suggested that day-7 is an adequate end point for setting the reference for characterizing the effects of test ligands on HSC-e cells.

Related to Figure 5.
