# Supplementary material for: Intercellular network structure and regulatory motifs in the human hematopoietic system
Source: Mol Syst Biol. 2014 Jul 15;10(7):741. doi: 10.15252/msb.20145141 (PMC4299490; doi:10.15252/msb.20145141)
Supplement: Supplementary file 7 — Supplementary Figure S7 [file msb0010-0741-sd7.pdf]

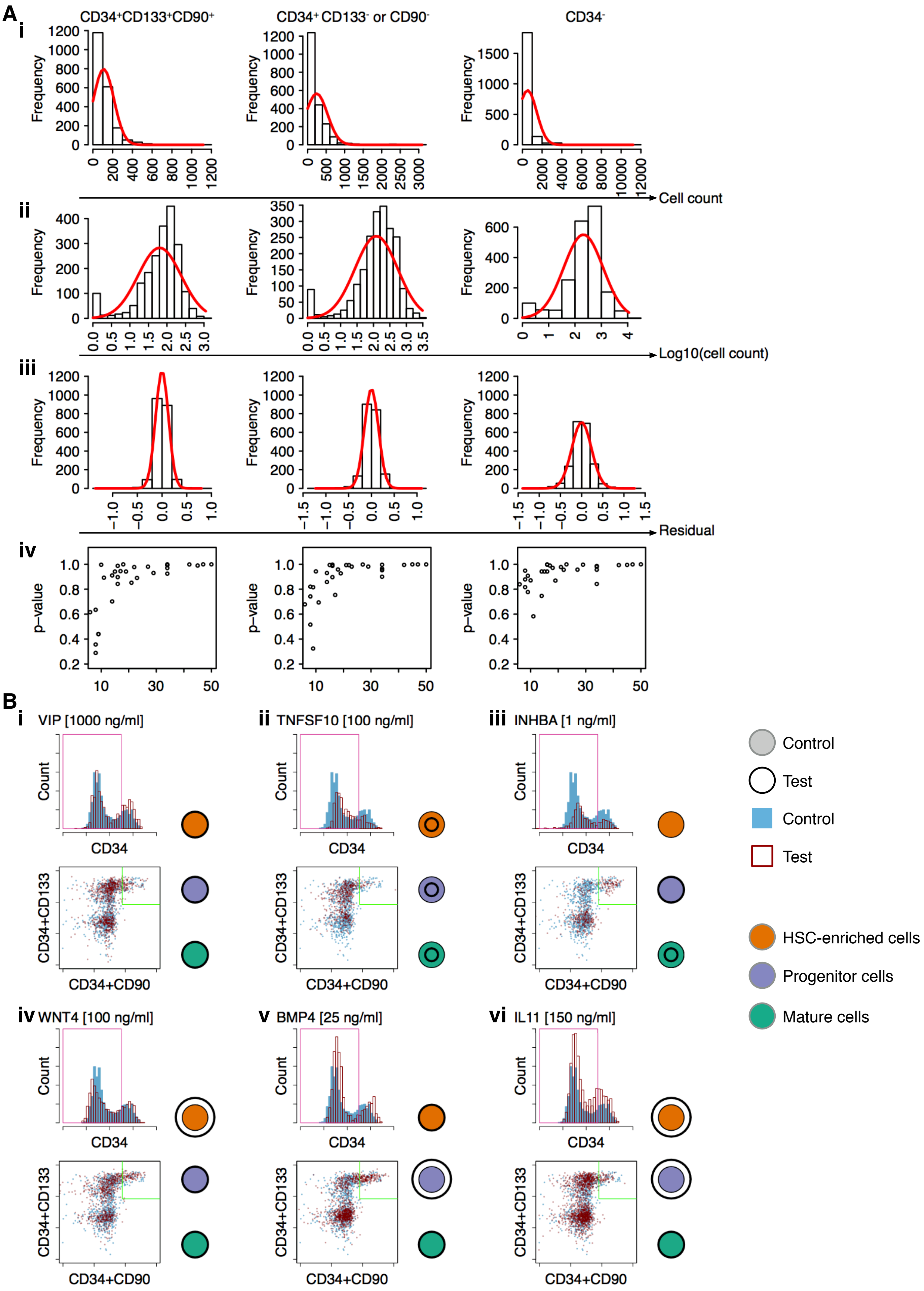

A
i
CD34+CD133+CD90+
CD34+ CD133- or CD90-
CD34-
Frequency
Cell count
ii
Log10(cell count)
iii
Residual
iv
p−value
B
i VIP [1000 ng/ml]
ii TNFSF10 [100 ng/ml]
iii INHBA [1 ng/ml]
iv WNT4 [100 ng/ml]
v BMP4 [25 ng/ml]
vi IL11 [150 ng/ml]
Count
CD34
CD34+CD133
CD34+CD90
Control
Test
Control
Test
HSC-enriched cells
Progenitor cells
Mature cells

Figure S7. Representative results of *in vitro* experiments for exogeneous ligands regulating HSC-e fate decisions.

A Data transformation and Fligner-Killeen test results for the *in vitro* ligand screening experiments. (i) Raw screening data are not normally distributed. The raw data are shown by bar plots, and fittings by normal distribution functions are shown in red. (ii) Log10 transformed raw data are normally distributed. The log10-transformed data are shown by bar plots, and fittings by normal distribution functions are shown in red. (iii) Residuals on the log10 scale are normally distributed. The residuals are shown by the bar plots, and fittings by normal distribution functions are shown in red. (iv) P-values of the Fligner-Killeen test versus the degree of freedom of the test. Each data point represents one experiment. Related to Figure 5.

B Representative results showing phenotypic effects of test ligands on $Lin^{-}Rho^{low}CD34^{+}CD38^{-}CD45RA^{-}$ $CD49f^{+}$ cells isolated from human umbilical cord blood samples. Blue: average results of the control conditions (N=4). (i) VIP (working concentration, WC = 1000 ng/ml) had no impact on any of the analyzed cell phenotypes comparing to the BC control (P-values were +0.3133, +0.1448 and -0.4914 for HSC-enriched cells, progenitor cells and mature cells, respectively). (ii) TNFSF10 (WC = 100 ng/ml) led to significant decrease in all three phenotypes (P-values were -0.0007, -0.0094 and -0.0207 for HSC-enriched cells, progenitor cells and mature cells, respectively). (iii) INHBA (Activin A, WC = 1 ng/ml) led to decrease in the number of mature cells (P-value = -0.0030) but no effect on the number of HSC-enriched cells (P-value = -0.0518) or progenitor cells (P-value = +0.3975). (iv) WNT4 (WC = 100 ng/ml) led to increase in the number of HSC-enriched cells (P-value = +0.0067) but no effect on the number of progenitor cells (P-value = +0.1694) or mature cells (P-value = +0.0995). (v) BMP4 (WC = 25 ng/ml) led to increase in the number of progenitor cells (P-value = +0.0053). (vi) IL11 (WC = 150 ng/ml) led to increase in the number of HSC-enriched cells (P-value = +0.0001) and progenitor cells (P-value = +0.0026). Brown: representative results of test ligands. The schematics next to the flow cytometry data represent differences in cell numbers (by circle sizes) between test conditions and the control condition. All the P-values were obtained using the nested ANOVA. Related to Figure 6.
