# Supplementary material for: Intercellular network structure and regulatory motifs in the human hematopoietic system
Source: Mol Syst Biol. 2014 Jul 15;10(7):741. doi: 10.15252/msb.20145141 (PMC4299490; doi:10.15252/msb.20145141)
Supplement: Supplementary file 8 — Supplementary Figure S8 [file msb0010-0741-sd8.pdf]

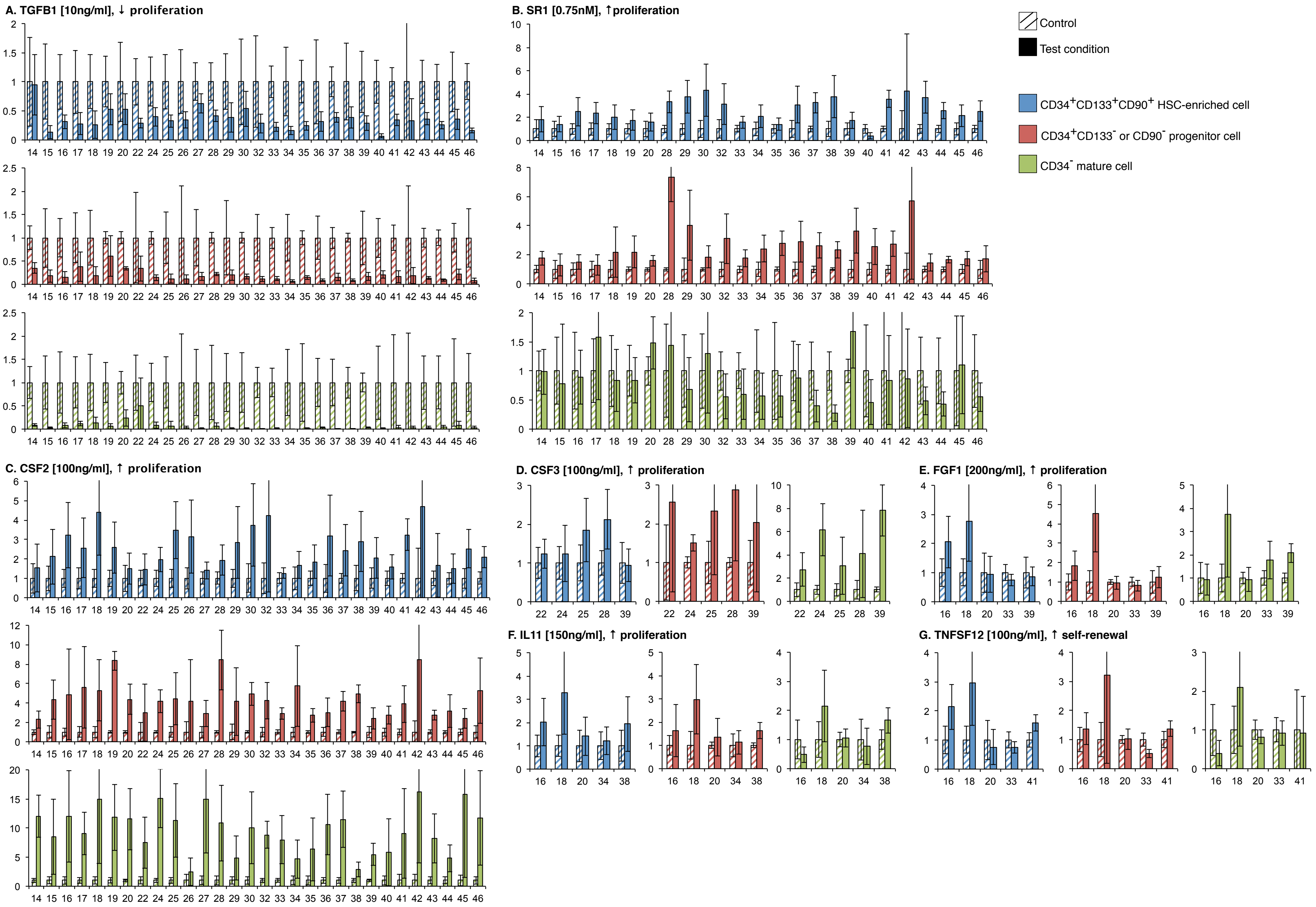
A. TGFB1 [10ng/ml], ↓ proliferation
B. SR1 [0.75nM], ↑proliferation
Control
Test condition
CD34+CD133+CD90+ HSC-enriched cell
CD34+CD133- or CD90- progenitor cell
CD34- mature cell
C. CSF2 [100ng/ml], ↑ proliferation
D. CSF3 [100ng/ml], ↑ proliferation
E. FGF1 [200ng/ml], ↑ proliferation
F. IL11 [150ng/ml], ↑ proliferation
G. TNFSF12 [100ng/ml], ↑ self-renewal

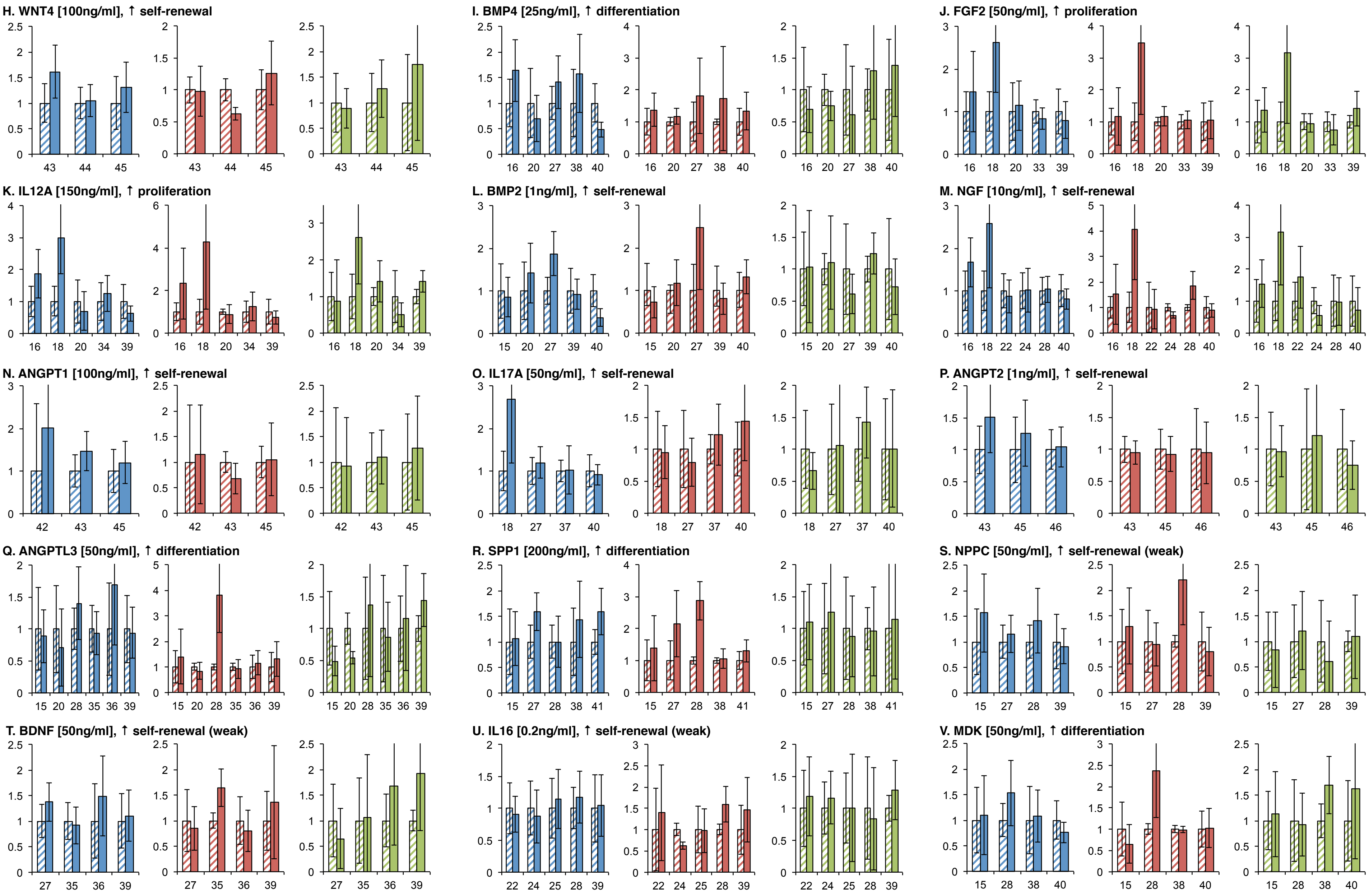
H. WNT4 [100ng/ml], ↑ self-renewal
43 44 45
I. BMP4 [25ng/ml], ↑ differentiation
16 20 27 38 40
J. FGF2 [50ng/ml], ↑ proliferation
16 18 20 33 39
K. IL12A [150ng/ml], ↑ proliferation
16 18 20 34 39
L. BMP2 [1ng/ml], ↑ self-renewal
15 20 27 39 40
M. NGF [10ng/ml], ↑ self-renewal
16 18 22 24 28 40
N. ANGPT1 [100ng/ml], ↑ self-renewal
42 43 45
O. IL17A [50ng/ml], ↑ self-renewal
18 27 37 40
P. ANGPT2 [1ng/ml], ↑ self-renewal
43 45 46
Q. ANGPTL3 [50ng/ml], ↑ differentiation
15 20 28 35 36 39
R. SPP1 [200ng/ml], ↑ differentiation
15 27 28 38 41
S. NPPC [50ng/ml], ↑ self-renewal (weak)
15 27 28 39
T. BDNF [50ng/ml], ↑ self-renewal (weak)
27 35 36 39
U. IL16 [0.2ng/ml], ↑ self-renewal (weak)
22 24 25 28 39
V. MDK [50ng/ml], ↑ differentiation
15 28 38 40

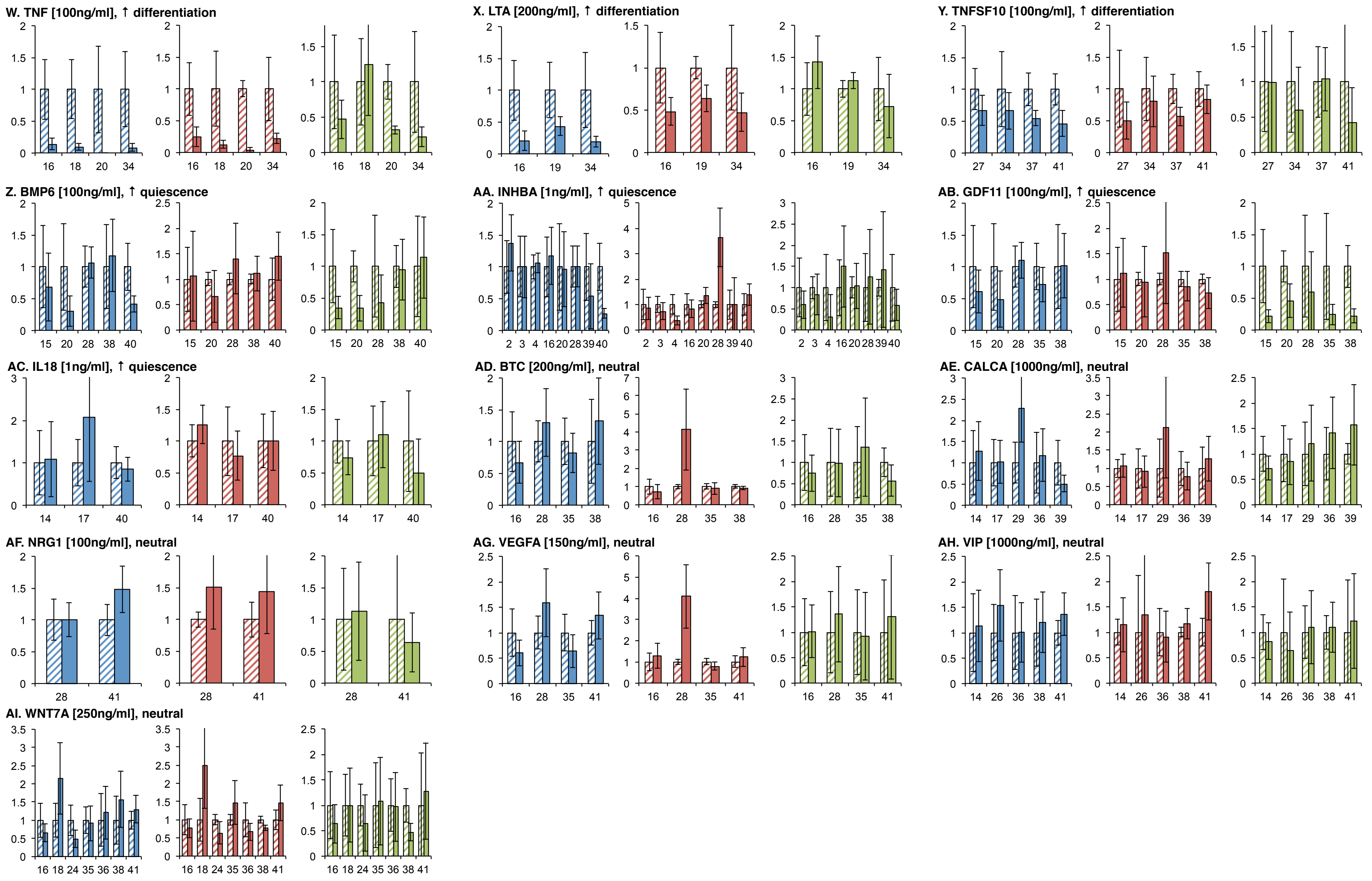

Figure S8. Normalized cell counts separated in each experiment. For each experiment, data of test conditions were normalized to data of the basic control condition.

Blue: $CD34^{+}CD90^{+}CD133^{+}$ HSC-enriched cells. Red: $CD34^{+}CD90^{-}$ or $CD133^{-}$ progenitor cells. Green: $CD34^{-}$ mature cells.
