# Supplementary material for: Intercellular network structure and regulatory motifs in the human hematopoietic system
Source: Mol Syst Biol. 2014 Jul 15;10(7):741. doi: 10.15252/msb.20145141 (PMC4299490; doi:10.15252/msb.20145141)
Supplement: Supplementary file 9 — Supplementary Figure S9 [file msb0010-0741-sd9.pdf]

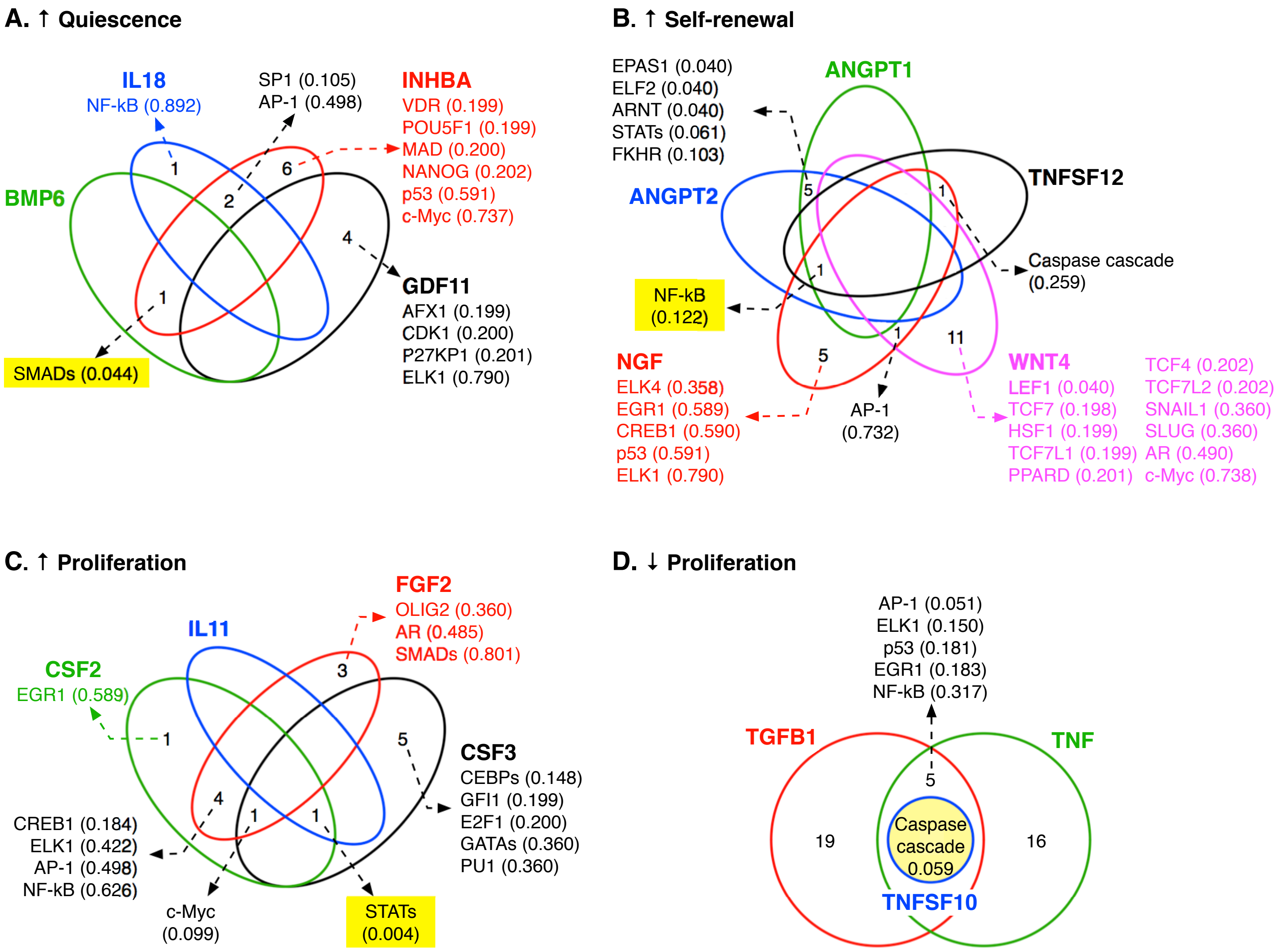

Figure S9. Ligands that direct HSC-e fate along different decision pathways are enriched in different intracellular pathway nodes.

A Intracellular pathway nodes of ligands that induced HSC-e quiescence. The numbers of overlaps between ligands are labeled. The unlabeled portions indicate no overlaps. Pathway nodes are labeled along with P-values obtained from a permutation test. The intracellular regulatory motif is highlighted in yellow.

B Intracellular pathway nodes of ligands that induced HSC-e self-renewal.

C Intracellular pathway nodes of ligands that induced HSC-e proliferation.

D Intracellular pathway nodes of ligands that inhibited HSC-e proliferation.

Related to Figure 7.
