# Supplementary material for: Intercellular network structure and regulatory motifs in the human hematopoietic system
Source: Mol Syst Biol. 2014 Jul 15;10(7):741. doi: 10.15252/msb.20145141 (PMC4299490; doi:10.15252/msb.20145141)
Supplement: Supplementary file 14 — Supplementary Table S5 [file msb0010-0741-sd14.pdf]

Hyper-geometric Z-score >1.15

Function	Pathway names	Hypergeometric Z-scores												
		HSCe	CMP	GMP	MEP	MLP	Mono	Neut	Baso	Eos	Mega	PreB	EryB	Others
Proliferation	Positive regulation of cell proliferation	-0.27	0.04	-0.98	-0.37	-1.46	-0.23	-0.88	-2.56	-1.61	-1.03	-1.69	0.90	-2.08
Survival	Survival (Anti-Apoptosis) mediated by external signals via PI3K-AKT	-2.04	-1.32	-0.06	-0.33	-1.47	0.15	-2.11	-1.66	-1.30	-0.48	-1.10	-0.51	-2.62
Survival	Survival (Anti-Apoptosis) mediated by external signals via MAPK and JAK-STAT	0.85	-0.85	0.38	0.94	-1.30	0.50	0.01	-1.21	-1.01	-0.74	-0.95	-1.10	-0.84
Cell adhesion	Leucocyte chemotaxis	-0.34	-0.03	-1.62	-1.11	-0.90	-1.51	-0.94	-2.16	-1.64	-1.56	-2.25	-2.94	1.32
Survival	Survival (Anti-apoptosis) mediated by external signals via NF-kB	-1.58	-0.93	-0.57	-0.16	-1.35	2.15	1.43	-1.30	1.12	0.42	-1.03	-1.22	-0.33
Development	Regulation of angiogenesis	-0.69	-1.46	-1.06	0.22	0.50	1.95	1.15	-1.34	-0.40	-1.59	-0.39	-2.01	-1.71
Proliferation	Negative regulation of cell proliferation	-1.74	-0.46	0.01	0.58	-0.67	-0.67	1.04	-0.31	0.86	0.02	0.07	0.11	-1.26

Inflammation	Innate inflammatory response	-0.96	-1.08	-0.70	0.66	-0.61	-0.61	0.44	-0.20	1.97	0.75	1.53	-2.00	-1.75
Survival	Survival inhibition (Apoptosis stimulation) by external signals	-1.09	-1.88	-0.83	-1.30	0.85	1.63	2.98	0.78	1.76	0.47	-0.68	-0.03	-1.36
Cell cycle	G1-S Growth factor regulation	-0.48	0.03	-0.14	-1.33	0.75	-0.02	-1.87	-0.52	-0.25	0.90	-0.13	1.41	-2.00
Cell cycle	G2-M	-0.87	-0.91	-0.77	-0.63	0.79	-0.74	-0.88	-1.07	-0.57	1.06	-0.96	0.76	-0.11
Cell cycle	G1-S Interleukin regulation	-0.95	-1.00	0.50	0.90	0.59	0.59	1.47	-0.12	-0.63	-0.26	1.24	1.41	-1.32
Immune response	T helper cell differentiation	-0.57	0.14	0.60	1.16	-0.24	-1.21	-1.44	1.17	2.65	0.90	3.18	-1.50	0.77

Pathway names	Pathway elements
Positive regulation of cell proliferation	BTC, CCL14, CLEC11A, COL18A1, CSF1, CSF2, CSF3, CTF1, CXCL10, CXCL5, EDN1, EGF, FGF10, FGF18, FGF2, FGF4, FGF7, FGF9, FLT3LG, HGF, IGF1, IL11, IL3, IL5, IL9, KITLG, LIF, PGF, PTN, PYY, SHH, TDGF1, VEGFA, VEGFB,
Survival (Anti-Apoptosis) mediated by external signals via PI3K-AKT	BDNF, CCL3, CLCF1, CNTF, CSF2, CSH2, EGF, FGF1, FGF10, FGF16, FGF19, FGF2, FGF3, FGF4, FGF6, FGF7, FGF8, FGF9, FIGF, FLT3LG, FN1, GDNF, HGF, IGF1, IL15, IL1B, IL2, IL4, IL7, IL8, INS, LAMA1, MICB, NGF, NRG1, NTF3, NTF4, PDGFA, PDGFB, POMC, PRL, THPO, ULBP1,
Survival (Anti-Apoptosis) mediated by external signals via MAPK and	ADCYAP1, CSF2, CSH2, EPO, FLT3LG, IL15, IL7, NGF, PDGFA, PDGFB, PDGFC, PDGFD, POMC, PRL, THPO

Leucocyte chemotaxis	CCL1, CCL11, CCL13, CCL14, CCL15, CCL16, CCL17, CCL18, CCL19, CCL2 CCL20, CCL21, CCL22, CCL23, CCL24, CCL25, CCL26, CCL3, CCL3L1 CCL4, CCL5, CCL7, CX3CL1, CXCL1, CXCL10, CXCL11, CXCL12, CXCL13, CXCL16, CXCL2, CXCL3, CXCL5, CXCL6, CXCL9, IL8, XCL1
Survival (Anti-apoptosis) mediated by external signals via NF-kB	CD40LG, CSF2, FN1, IL15, NGF, PDGFA, PDGFB, TNF, TNFSF11,, TNFSF12, TNFSF13,, TNFSF13B, TNFSF15,, TNFSF4,,TNFSF8, VEGFA
Regulation of angiogenesis	AGT, ANGPT1, CCL2, CTGF, DHH, EDN1, EFNA1, EFNA5, EFNB1, EFNB2, HBEGF, IHH, ,IL15, IL18, IL1A, IL1B, IL6, IL8, LEP, PF4, SHH, TGFB2, TNFSF12, VEGFA
Negative regulation of cell proliferation	CCL23, CCL3L1, COL18A1, CXCL1, GDF11, GNRH1, IFNB1, IFNG, IFNK, IGF1, IL1A, IL1B, IL6, IL8, OSM, PTHLH, SST,
Innate inflammatory response	C4A, C5, CCL20, DEFB1, IFNA1, IFNB1, IL18, IL1A, IL1B, IL36A, IL36B, IL36G, IL36RN, IL37, IL4, IL6, IL8, TNF
Survival inhibition (Apoptosis stimulation) by external signals	BDNF, CD70, FASLG, HBEGF, INHBA, LEP, LTA, LTB, NGF, NRG1, NTF3, NTF4, TGFB1, TGFB2, TGFB3, TNF, TNFSF10, TNFSF12, TNFSF14, TNFSF18
G1-S Growth factor regulation	BTC, EGF, FGF1, FGF2, FGF3, FGF6, FGF8, FGF9, HGF, IGF1, IGF2, INHA, INHBA, LTBP3, PDGFA, TGFA, TGFB1, TGFB2, TGFB3, TNFSF15, VEGFA
G2-M	EGF, IGF1, IGF2, PDGFA, PDGFB
G1-S Interleukin	IL10, IL1A, IL1B, IL2, IL22, IL4
T helper cell differentiation	CD40LG, IFNB1, IFNG, IL12A, IL12B, IL13, IL17A, IL18, IL2, IL21, IL4, IL6, TNFSF9
