# Supplementary material for: Intercellular network structure and regulatory motifs in the human hematopoietic system
Source: Mol Syst Biol. 2014 Jul 15;10(7):741. doi: 10.15252/msb.20145141 (PMC4299490; doi:10.15252/msb.20145141)
Supplement: Supplementary file 19 — Supplementary Information S1 [file msb0010-0741-sd19.docx]

**SUPPLEMENTARY INFORMATION**

**Optimization of *in vitro* experiments**

Human Lin^-^ cells were isolated from umbilical cord blood (UCB) samples and stained with the following antibodies (all from BD Biosciences, Mississauga, ON, Canada) in 1:100, unless otherwise stated: Rhodamine (Invitrogen R302, Grand Island, NY, US), CD34 (APC), CD38 (PE-Cy7), CD45RA (eFluor 405, eBiosciences, San Diego, CA, USA), CD49f (PE, 1:25). Forty Lin^-^Rho^low^CD34^+^CD38^-^CD45RA^-^CD49f^+^ cells were sorted, using the same gating strategies described at (Notta *et al*, 2011), and dispensed per well in a 96-well V-bottom plate with a MoFloXDP flow cytometer (Beckman Coulter).

The high content screening assay was optimized in terms of basal cytokines (BC) and culture time. Due to low frequency of Lin^-^Rho^low^CD34^+^CD38^-^CD45RA^-^CD49f^+^ cells in human UCB samples, we decided to seed 40 cells on day 0. As can be seen from Figure S6A, the total cell numbers decreased significantly without cytokine supplementation or when SCF (100 ng/ml), THPO (50 ng/ml) and FLT3LG (100 ng/ml) were added individually. Supplementing the cell culture with a cocktail of SCF, THPO and FLT3LG increased the total cell number by 17.6±10.6 fold total cell expansion in 7 days (Figure S6B). This allowed for automated analysis of cell outputs using an LSRFortessa flow cytometer (BD Biosciences, San Jose, CA, USA). Thus, we decided to use the cocktail of SCF (100 ng/ml), THPO (50 ng/ml) and FLT3LG (100 ng/ml) to test the effects of the ligands of interest on human UCB Lin^-^Rho^low^CD34^+^CD38^-^CD45RA^-^CD49f^+^ cells.

Next, we sought to optimize the culturing time by culturing the cells for 7, 10 and 12 days, and then comparing the distribution of CD34^+^CD90^+^CD133^+^ HSC-enriched cells, CD34^-^ cells (defined as mature cells) and CD34^+^ cells that are CD133^-^ or CD90^-^ (defined as progenitor cells). As can be seen from Figure S6C, almost all the cells in culture were mature cells by day 10 [(0.03±0.06)% HSC-enriched cells, (0.26±0.35)% progenitor cells and (99.71±0.31)% mature cells], whereas on day 7, there were (6.35±3.21)% HSC-enriched cells, (27.75±6.86)% progenitor cells and (65.90±10.04)% mature cells. Distribution of the 3 populations would allow us to evaluate if the ligand of interest has effect on any of the 3 populations in combination of the BC cocktail.

In the optimized protocol, the cells were cultured in media consisted of 100μl serum-free media containing IMDM (GIBCO, Rockville, MD), 20% BIT serum substitute (StemCell Technologies), 1% Penn/Strep (GIBCO) and 1% Glutamax (GIBCO). The culture media was supplemented with 100 ng/ml SCF, 100 ng/ml FLT3LG and 50 ng/ml THPO, 1 μg/ml low-density lipoproteins (LDL, Calbiochem, La Jolla, CA, USA), and a test ligand at concentrations shown in Table E7. All the ligands were from R&D Systems, USA. On day 7, cells were stained with the following antibodies (all from BD in 1:100): CD34 (FITC), CD133 (PE) and CD90 (APC). Total cell counts (N_Total_’), CD34^+^CD133^+^CD90^+^ cell (HSC-enriched cell, N_HSC-enriched_’) counts and CD34^-^ cell (mature cells, N_Mature_’) counts were obtained using an LSRFortessa flow cytometer (BD Bioscience). The flow cytometer had a dead volume of 30μl. To take into account the cells in the deal volume, we calculated N_Total_, N_HSC-enriched_’ and N_Mature_’ using the following equations:

$\text{N}_{\text{Total}}\text{ = }{\text{N}_{\text{Total}}}^{\text{'}}\text{ * }\frac{\text{V}_{\text{Analyze}}\text{ }\text{+}\text{ }\text{30}}{\text{V}_{\text{Analyze}}}$,

$\text{N}_{\text{HSC-enriched}}\text{ = }{\text{N}_{\text{HSC-enriched}}}^{\text{'}}\text{ * }\frac{\text{V}_{\text{Analyze}}\text{ }\text{+}\text{ }\text{30}}{\text{V}_{\text{Analyze}}}$,

$\text{N}_{\text{Mature}}\text{ =}{\text{ N}_{\text{Mature}}}^{\text{'}}\text{ * }\frac{\text{V}_{\text{Analyze}\text{ }}\text{+}\text{ }\text{30}}{\text{V}_{\text{Analyze}}}$,

where V_Analyze_ is the analyzed volume (120 μl) by flow cytometry. Progenitor cell number was calculated as N_Total_ – N_HSC-enriched_ – N_Mature_, where N_Total_, N_HSC-enriched_ and N_Mature_ represent cell counts of total, HSC-enriched and mature cells, respectively.

**Statistical analysis for *in vitro* experiments**

An *in vitro* high content screening was performed to test the effects of 40 cytokines (3 concentrations per cytokine) and 5 soluble inhibitors (1 concentration per inhibitor) on human umbilical cord blood-derived Lin^-^Rho^low^CD34^+^CD38^-^CD45RA^-^CD49f^+^ HSC-enriched cells were tested. Including the 4 control conditions [no cytokine supplement, basal cytokine (BC)-supplemented condition, BC + SR1, and BC + TGFB1], there were 40*3+5*1+4=129 treatments in total (each ligand and concentration combination is called one treatment). For each treatment, the number of CD34^+^CD90^+^CD133^+^ HSC-enriched cells, CD34^-^ cells (defined as mature cells) and CD34^+^ cells that are CD133^-^ or CD90^-^ (defined as progenitor cells) were counted using flow cytometry on day 7.

The 40 cytokines and 5 soluble inhibitors were split into a number of experiments. Each experiment included the 4 controls to check if the experiment was functional, and 3 technical replicates per treatment. For each treatment, there were at least 3 biological replicates. The goal of this study is to test if any treatment (i.e., cytokine/inhibitor and concentration combination) affected output of CD34^+^CD90^+^CD133^+^ HSC-enriched cells, progenitor cells and mature cells comparing to the BC control condition. The data were analyzed using the mixed model nested ANOVA in R. Below is the analysis method. The R scripts for the analysis are available upon request.

1. Data transformation

ANOVA assumes that (1) data are normally distributed, and (2) group variances are equal. In our data, the raw absolute cell numbers were not normally distributed, as shown in Figure S7A-i as observed in many biology data (Limpert *et al*, 2001). The data were first log10() transformed. Because some data points were 0, a constant of 1 was added to all the raw data before transformation. The data were close to normal distribution after the transformation (Figure S7A-ii and Figure S7A-iii).

1. Homogeneous variance for data from individual experiment

After data transformation, variance among treatments was compared for each experiment. Variance homogeneity was tested using the Fligner-Killeen (median) test in R. Figure S7A-iv shows the p-values from the Fligner-Killeen test. The results show that there is no evidence (P-value > 0.05) to against the null hypothesis that the data have homogeneous variance, i.e., treatments in each experiment had equal variance.

1. Equal variance of the basic control across experiments

Equal variation of the basic control condition across experiments was tested using the Fligner-Killeen test. The results (Box 1) show that there is no evidence (P-value > 0.05) to against the null hypothesis that the results of the basic condition are equally variable across experiments.

Box 1. Results of the Fligner-Killeen test for variance of the basic control condition across experiments.


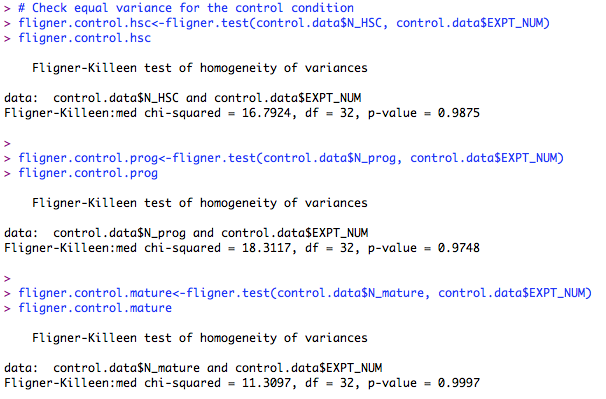


1. Unequal variances across experiments

Equal variance across experiments was tested using the Fligner-Killeen test. The results (Box 2) show that there is significantly strong evidence (P-value < 0.0001) to against the null hypothesis that the data across experiments are equally variable.

Box 2. Results of the Fligner-Killeen test for variance across experiments.


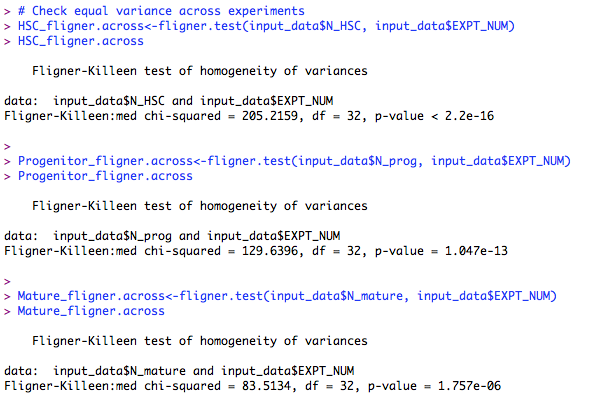


1. Source of the observed unequal variance across experiments

Nest, we tested if the observed variance across experiments (Box 2) is due to different treatments. Two models were constructed: Model 1 assumes that the measured variables did not depend on treatment, and Model 2 assumes that the measured variables depend on treatments. The results from comparing the two models suggested that treatments have significant contribution (P-value < 0.0001) to the observed variation (Box 3).

Box 3. Results of the Fligner-Killeen test for variance across experiments.


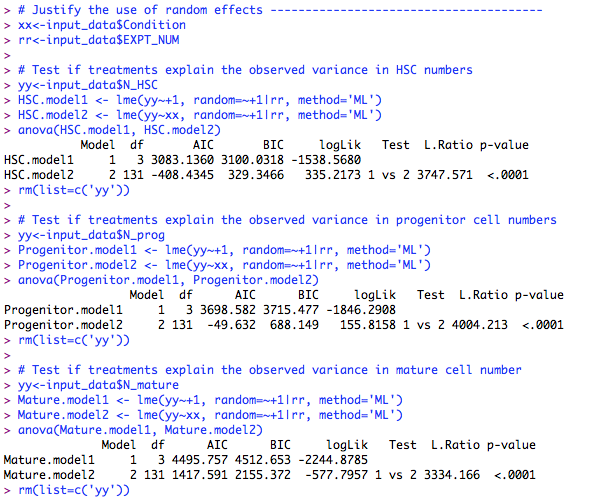


1. Contrast treatments against controls

To compare any of all the other treatments to the basic control condition, a linear mixed-model was constructed for each treatment, and compared to that of the basic control condition. The model was constructed using the lme() function in the nlme package in R. This function allows unequal number of samples per group. The random effect, experiment date was nested within the fixed effect, cell culture treatments. This allows the measured cell numbers to vary randomly between experiments. Log-likelihood was maximized in model fitting. The models of total cell numbers, HSC cell numbers, progenitor numbers and mature cell numbers are following:

TNC.mod<-lme(tnc~treatment, random = ~1|expt_date, method = ‘ML’)

HSC.mod<-lme(hsc~treatment, random = ~1|expt_date, method = ‘ML’)

Progenitor.mod<-lme(progenitor~treatment, random = ~1|expt_date, method = ‘ML’)

Mature.mod<-lme(mature~treatment, random = ~1|expt_date, method = ‘ML’)

Below are the R packages used for the analyses,


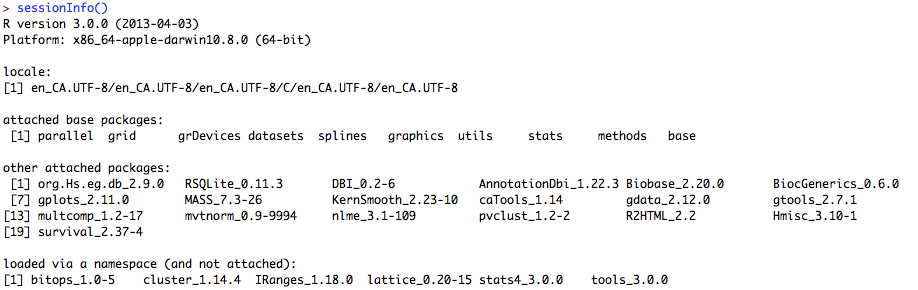


**References**

Limpert E, Stahel W. & Abbt M (2001) Log-normal Distributions across the Sciences: Keys and Clues. *Bioscience* **51:** 341–352

Notta F, Doulatov S, Laurenti E, Poeppl A, Jurisica I & Dick JE (2011) Isolation of single human hematopoietic stem cells capable of long-term multilineage engraftment. *Science.* **333:** 218–21
