# Supplementary material for: Intercellular network structure and regulatory motifs in the human hematopoietic system
Source: Mol Syst Biol. 2014 Jul 15;10(7):741. doi: 10.15252/msb.20145141 (PMC4299490; doi:10.15252/msb.20145141)
Supplement: Supplementary file 20 — Supplementary Table Legends [file msb0010-0741-sd20.docx]

**SUPPLEMENTARY TABLE LEGENDS**

**Table S1. Thirteen hematopoietic gene sets.** Related to Figure 2.

**Table S2. 933 ligand-receptor pairs compiled from iRefWeb database.** Related to Figure 2. See also Materials and Methods.

**Table S3. Differentially over-expressed (A) ligand and (B) receptor genes identified at a false discovery rate of 10%.**

**Table S4. Constructed cell-cell communication networks at a false discovery rate of 10% for definition of differentially over-expressed ligand and receptor genes.**

**Table S5. Function associated ligand sets.**

**Table S6. Hypergeometric Z-scores shown in Figure 3C.**

**Table S7. Literature on 33 HSCe-targeting ligands.**

**Table S8. Raw results of *in vitro* experiments.**

Shown are absolute cell numbers. Ligands of interest were tested in the presence of basal cytokines including 100 ng/ml SCF, 50 ng/ml THPO, and 100 ng/ml FLT3LG. HSC-enriched cells: CD34^+^CD133^+^CD90^+^. Progenitor cells: CD34^+^ cells that are CD90^-^ or CD133^-^. Mature cells: CD34^-^.

**Table S9. P-values from the nested ANOVA test and functional categorization for the ligands of interest.**

Functional categorization for the ligands of interest in terms of exogenously manipulating fate decisions of Lin^-^Rho^low^CD34^+^CD38^-^CD45RA^-^CD49f^+^ HSC-enriched cells isolated from human umbilical cord blood. The signs of P-values shows the directionalit: positives indicate that results with the ligand of interest are higher than the basal control, and negative indicate that results of the ligand of interest are lower than the basal control. The nested ANOVA was performed on log10 transformed data. The highlighted results are shown in Figure 5C and Figure 6A.
